# Third-window anomalies in children: an imaging-based educational review

**DOI:** 10.1007/s00247-026-06693-0

**Published:** 2026-06-16

**Authors:** Melike Zeynep Akış, Fatma Ceren Sarıoğlu, Serhat Akış, Cemresu Akın, Enis Alpin Güneri, Handan Güleryüz Uçar

**Affiliations:** 1 Department of Radiology, Dokuz Eylül University, Izmir, Turkey; 2 Department of Radiology, Izmir Tepecik Eğitim ve Araştırma Hastanesi, Izmir, Turkey; 3 Department of Otolaryngology–Head and Neck Surgery, Dokuz Eylül University, Izmir, Turkey

**Keywords:** Enlarged vestibular aqueduct, Pediatric inner ear, Semicircular canal dehiscence, Temporal bone computed tomography, Third-window anomalies

## Abstract

Third-window anomalies represent a heterogeneous group of inner ear disorders characterized by abnormal openings in the bony otic capsule, resulting in altered cochlear and vestibular fluid mechanics. By creating an alternative pathway for inner ear fluid motion due to a new opening in addition to the oval and round windows, these lesions disrupt normal sound transmission and vestibular function. In pediatric patients, recognition of third-window anomalies is particularly important, as early and accurate diagnosis supports appropriate clinical assessment and management. High-resolution computed tomography of the temporal bone is the primary imaging modality for evaluating third-window anomalies, enabling detailed assessment of the bony labyrinth. However, many of these abnormalities are subtle and may be underestimated or misinterpreted on standard axial images. The use of anatomy-oriented multiplanar reconstructions tailored to the suspected defect significantly improves lesion conspicuity and diagnostic confidence. This educational review summarizes the anatomic and pathophysiologic principles underlying third-window phenomena in children and illustrates the imaging features of major entities, including semicircular canal dehiscence, enlarged vestibular aqueduct, and less common cochlear dehiscence. The aim is to provide a clear and practical imaging-based framework to support radiologists involved in the evaluation of pediatric inner ear abnormalities.

**Graphical Abstract:**

**Figure:**
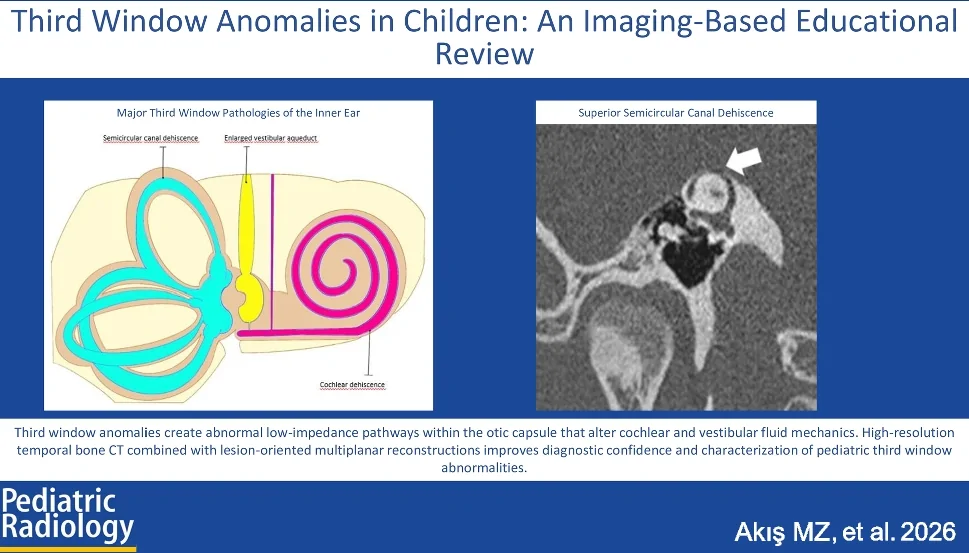

## Introduction

The inner ear is enclosed within the dense bony otic capsule of the temporal bone and consists of the cochlea, which mediates hearing, and the vestibular system, which is responsible for balance [1, 2]. The cochlea converts sound-induced motion of inner ear fluids into neural signals, whereas the vestibule and semicircular canals detect linear and angular acceleration through motion of inner ear fluids [3].

Normal sound transmission within the inner ear depends on the coordinated function of the oval and round windows [4–6]. Vibratory motion of the stapes at the oval window generates pressure waves within the perilymphatic spaces of the cochlea, which propagate through the scala vestibuli and scala tympani [4, 6]. Because inner ear fluids are functionally incompressible, inward displacement at the oval window is accompanied by compensatory outward motion of the round window membrane, allowing bulk fluid movement within the cochlea [4–6]. This coordinated motion establishes pressure differences across the cochlear partition, driving basilar membrane motion and enabling sound perception [4–6].

In addition to the oval and round windows, the inner ear contains several small anatomic pathways that connect the perilymphatic spaces to surrounding structures, including the vestibular aqueduct, cochlear aqueduct, and small foramina transmitting vascular or neural elements [7, 8]. Under normal conditions, these pathways are narrow and elongated and are associated with high acoustic impedance, resulting in minimal contribution to sound transmission [7–9]. In this context, such structures have been described as physiologic or normal third windows [1].

Pathologic third-window anomalies arise when defects or marked thinning develop within the bony otic capsule, creating abnormal additional openings between the inner ear and adjacent compartments [1, 10]. These openings act as low-impedance pathways that divert acoustic energy and alter normal cochlear and vestibular fluid mechanics [1, 10, 11]. Depending on their anatomic location, pathologic third windows may involve the semicircular canals, vestibule, or cochlea [1, 10].

With the increasing use of high-resolution temporal bone computed tomography, a broad spectrum of third-window anomalies has been recognized, including superior, posterior, and lateral semicircular canal dehiscence, enlarged vestibular aqueduct, cochlear dehiscence, and abnormalities involving the internal auditory canal [2, 10–14]. In pediatric patients, these abnormalities are frequently congenital and may coexist with developmental inner ear malformations, making accurate, anatomy-oriented imaging assessment particularly important [2, 14].

The purpose of this educational review is to provide a comprehensive overview of third-window anomalies in children, grounded in established anatomic and physiologic principles, and to highlight key imaging features and diagnostic considerations using high-resolution temporal bone computed tomography.

## Normal inner ear anatomy and sound transmission

The inner ear is located within the petrous portion of the temporal bone and is enclosed by the dense bony otic capsule, which provides the rigid framework required for normal auditory and vestibular function [1, 2]. It consists of the cochlea, responsible for hearing, and the vestibular system, comprising the vestibule and the three semicircular canals, which mediate balance and spatial orientation [1–3].

**Fig. 1:**
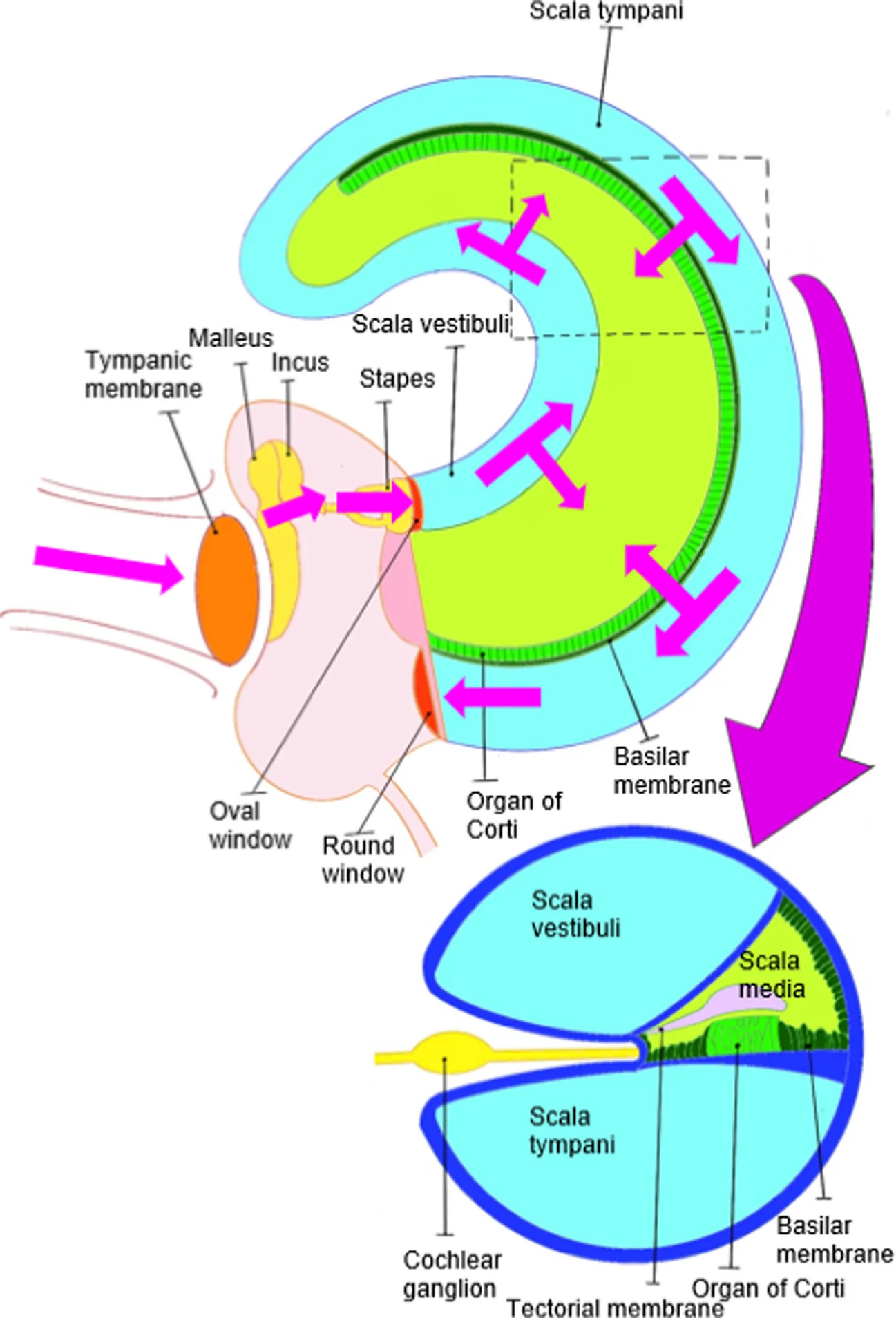
Normal sound transmission within the inner ear. Schematic anatomic illustration of normal middle-to-inner ear sound transmission. Sound-induced vibration of the stapes at the oval window transmits pressure into the perilymphatic spaces of the cochlea, with propagation of pressure waves through the scala vestibuli and scala tympani toward the cochlear apex. The diagram highlights the anatomic relationship between the oval window, round window, cochlear scalae, basilar membrane, and organ of Corti, which together form the structural basis for normal cochlear transduction

Normal sound transmission within the inner ear depends on the coordinated interaction between the oval and round windows, which function as the primary and secondary acoustic windows of the cochlea [4–6]. Vibratory motion of the stapes at the oval window generates pressure waves within the perilymphatic spaces of the cochlea. Because inner ear fluids are functionally incompressible, inward displacement at the oval window is accompanied by compensatory outward motion of the round window membrane, allowing bulk fluid movement and establishing the pressure gradients necessary for cochlear transduction [4–6]. This sequence of middle-to-inner ear sound transmission is schematically illustrated in Fig. 1.

In addition to the oval and round windows, several small anatomic channels connect the inner ear fluid spaces to adjacent compartments, including the vestibular aqueduct, cochlear aqueduct, and small foramina transmitting vascular or neural structures [7–9]. Under normal conditions, these pathways are narrow, elongated, and associated with high acoustic impedance, resulting in minimal contribution to sound transmission and preserved cochlear and vestibular fluid mechanics [1, 7–9].

## Concept of the third window

**Fig. 2:**
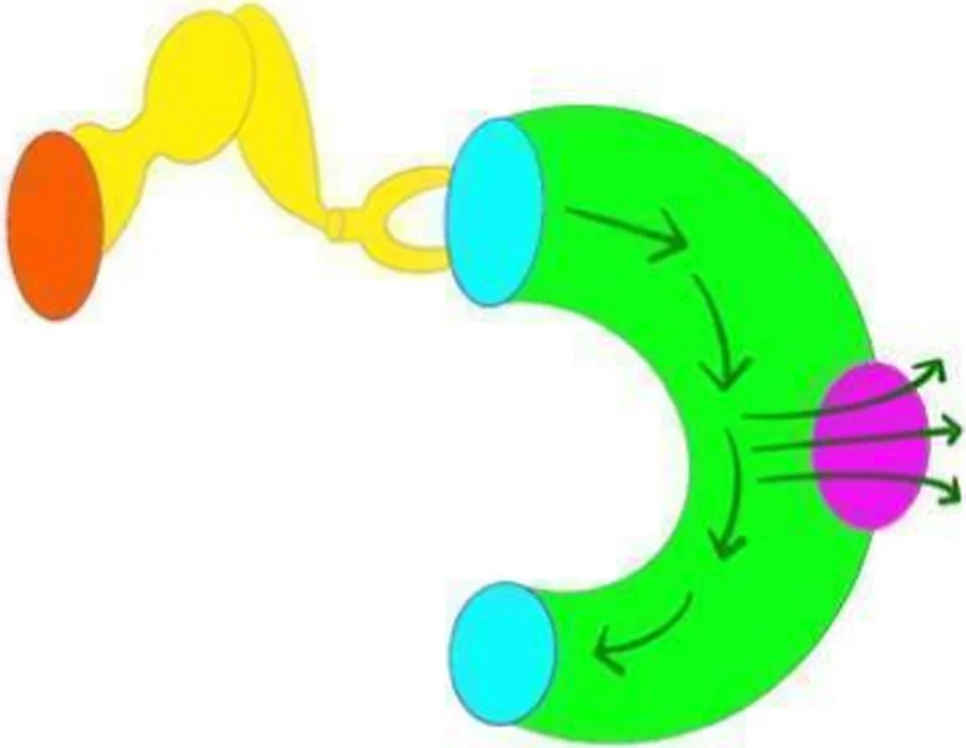
Schematic illustration of the third-window phenomenon. Under normal conditions, the oval and round windows (*blue*) form a closed hydraulic system within the inner ear, allowing efficient pressure transmission during sound stimulation. The presence of an additional abnormal opening in the bony otic capsule (*purple*) creates a low-impedance path for inner ear fluid motion, resulting in diversion of acoustic energy away from the cochlea and disruption of normal pressure gradients

A pathologic third window arises when a defect or marked thinning develops within the bony otic capsule, creating an abnormal additional opening that functions as a low-impedance pathway for inner ear fluid motion [1, 10–12]. In the presence of such a defect, the normally closed hydraulic system of the inner ear is disrupted, and part of the acoustic energy entering through the oval window is abnormally diverted through the third window rather than being fully transmitted throughout the cochlea. This shunting effect reduces the effective pressure gradient across the cochlear partition and diminishes cochlear stimulation, forming the fundamental mechanism underlying third-window-related auditory symptoms, as depicted in Fig. 2 [10–12].

**Fig. 3:**
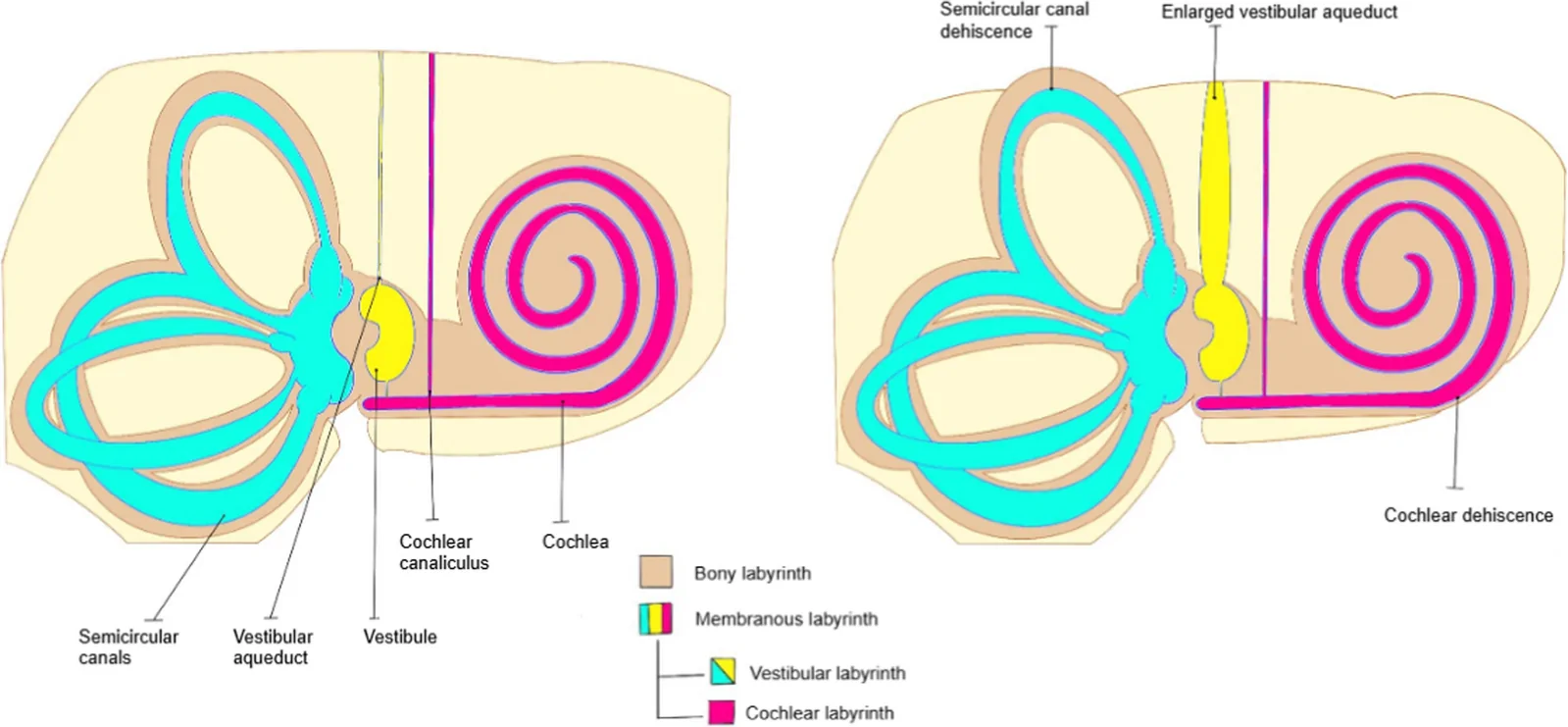
Comparative illustration of normal and pathologic inner ear anatomy. This dual-panel illustration contrasts normal inner ear anatomy (*left*) with representative third-window pathologies (*right*), including superior semicircular canal dehiscence, enlarged vestibular aqueduct, and cochlear dehiscence. These abnormalities create additional bony openings within the otic capsule, allowing aberrant fluid or pressure transmission that disrupts normal inner ear mechanics and contributes to audiovestibular symptoms

The anatomic spectrum of third-window anomalies is illustrated in Fig. 3, which contrasts with normal inner ear anatomy. As shown, abnormal bony openings involving different components of the inner ear—including the semicircular canals, vestibule, and cochlea—result in loss of the normal bony separation between inner ear fluid spaces and adjacent structures.

In pediatric patients, pathologic third-window anomalies most commonly reflect congenital or developmental abnormalities of the otic capsule and may coexist with other inner ear malformations, whereas acquired causes such as trauma or inflammatory erosion are less frequent [1, 2, 10–14]. A clear understanding of the third-window concept therefore provides a critical framework for interpreting imaging findings and correlating radiologic features with clinical manifestations in children.

## Classification of third-window anomalies

Third-window anomalies can be classified according to the anatomic site of disruption within the bony otic capsule and the specific inner ear compartment involved [1, 10]. From an anatomic and functional perspective, the inner ear is composed of three major components—the semicircular canals, the vestibule, and the cochlea—each of which plays a distinct role in auditory and vestibular mechanics [1–3]. Accordingly, third-window lesions are most practically categorized based on the inner ear structure affected by the abnormal bony opening [1, 10].

This anatomic classification provides a clear, imaging-based framework that is particularly useful in pediatric patients, in whom third-window anomalies are frequently congenital and may coexist with developmental inner ear malformations [2, 14]. The major categories of third-window anomalies and representative entities are summarized in Table 1.

**Table 1:** Anatomic classification of third-window anomalies

1. Semicircular canal	2. Vestibule	3. Cochlea
Superior canal dehiscence	Enlarged vestibular aqueduct	Inner ear malformations causing a dehiscence between cochlea and non-cochlear sites
Posterior canal dehiscence	Inner ear malformations causing a dehiscence between vestibule and non-vestibule sites	Cochlear–internal auditory canal dehiscence (e.g., DFNX2 [X-linked deafness with stapes gusher, IP-3 anomaly])
Lateral canal dehiscence		

## Clinical correlation

Third-window anomalies produce characteristic auditory and vestibular manifestations caused by abnormal shunting of acoustic energy and pressure through a low-impedance defect in the bony otic capsule. Auditory findings most commonly include apparent conductive or mixed hearing loss despite a normal middle ear, typically presenting as a low-frequency air-bone gap with normal tympanometry and preserved acoustic reflexes, reflecting altered cochlear mechanics rather than otosclerosis [1, 11]. Autophony, supra-normal bone conduction thresholds, and enhanced perception of internally generated sounds may also be observed due to abnormal vibration transmission through the third window [2, 10].

Vestibular manifestations vary according to the anatomic compartment involved and may include sound-induced vertigo (Tullio phenomenon), pressure-induced vertigo and/or nystagmus (Hennebert sign), oscillopsia, imbalance, and chronic disequilibrium, particularly in semicircular canal–related third-window anomalies [10–12]. In pediatric patients, these symptoms are frequently under-recognized, as children may be unable to clearly describe subjective sensations. Instead, presentation may be indirect, including imbalance, delayed motor development, avoidance of loud environments, or fluctuating hearing performance [2, 14].

As summarized in Table 2, the clinical phenotype correlates closely with the involved inner ear compartment. In cochlear third-window anomalies, recognition of abnormal low-impedance communications is also clinically relevant because such defects may have important surgical implications, including an increased risk of perilymph gusher during stapes or cochlear implant surgery [1, 15, 16]. Objective audiovestibular testing plays a complementary role in supporting the diagnosis and may demonstrate findings, such as abnormally low cervical vestibular evoked myogenic potential (c-VEMP) thresholds and preserved acoustic reflex patterns, in a way that is incompatible with conductive hearing losses or supra-normal bone conduction hearing thresholds [1, 11, 17–19].

**Table 2:** Predominant clinical manifestations of third-window anomalies according to the involved inner ear compartment

Inner ear compartment (third-window type)	Typical clinical manifestations	Predominant clinical domain
1. Semicircular canals (SSCD, PSCD, LSCD)	Apparent conductive or mixed hearing loss; sound-induced vertigo (Tullio phenomenon); pressure-induced vertigo and/or nystagmus (Hennebert sign); oscillopsia; imbalance [1, 2, 10–12, 27]	Vestibular ± auditory
2. Vestibule (EVA)	Fluctuating or progressive sensorineural/mixed hearing loss; episodic vertigo or imbalance; delayed motor development in children [2, 13]	Auditory > vestibular
3. Cochlea (cochlear third-window anomalies)	Severe sensorineural or mixed hearing loss; apparent conductive component without middle ear pathology; possible surgical implications [1, 2, 14, 15]	Auditory

## Imaging technique and role of lesion-oriented multiplanar reconstructions

High-resolution computed tomography (HRCT) is the reference standard for imaging evaluation of third-window anomalies because of its superior spatial resolution and ability to depict subtle osseous defects of the otic capsule [2, 18, 19]. Nevertheless, accurate diagnosis depends not only on thin-section acquisition but also on optimized imaging protocols and systematic use of lesion-oriented multiplanar reconstructions (MPRs), as many potential third-window sites are obliquely oriented relative to conventional imaging planes.

Accurate evaluation of third-window anomalies requires HRCT acquisition tailored to the complex anatomy of the otic capsule. Submillimeter collimation, with a recommended slice thickness of 0.5–0.625 mm, is essential to minimize partial-volume averaging and to ensure precise depiction of thin bony structures [2, 20]. A small field of view (approximately 16–22 cm), reconstruction with a high-frequency bone algorithm, and optimized bone window settings further enhance visualization of fine osseous details. Key CT acquisition parameters for optimal evaluation of third-window anomalies are summarized in Table 3.

**Table 3:** HRCT acquisition protocol for third-window anomalies

Parameter	Recommended technique	Rationale
Imaging modality	High-resolution computed tomography (HRCT)	Optimal depiction of subtle osseous defects of the otic capsule
Scan coverage	Temporal bones only	Reduces radiation exposure and avoids unnecessary scanning of the entire face or skull
Collimation/slice thickness	Submillimeter collimation; 0.5–0.625 mm	Minimizes partial-volume averaging and improves visualization of thin bony structures
Acquisition plane	Axial acquisition parallel to the lateral semicircular canal or infraorbitomeatal line	Provides standardized dataset suitable for high-quality multiplanar reconstructions
Field of view (FOV)	Small field of view (approximately 16–22 cm)	Maximizes in-plane spatial resolution
Reconstruction algorithm	High-frequency bone reconstruction kernel	Enhances edge definition and fine osseous details
Matrix size	High-resolution matrix (e.g., 512×512)	Improves spatial resolution for detailed temporal bone assessment
Tube voltage/current (pediatric)	Age- and body size–adjusted kVp and mAs	Optimizes radiation dose while maintaining diagnostic image quality
Contrast administration	Not routinely required	Third-window anomalies represent osseous defects rather than soft-tissue lesions
Emerging imaging technology	Photon-counting detector CT (PCD-CT)	Provides improved spatial resolution, reduced blooming and partial-volume artifacts, and potential pediatric dose advantages for temporal bone imaging

**Fig. 4:**
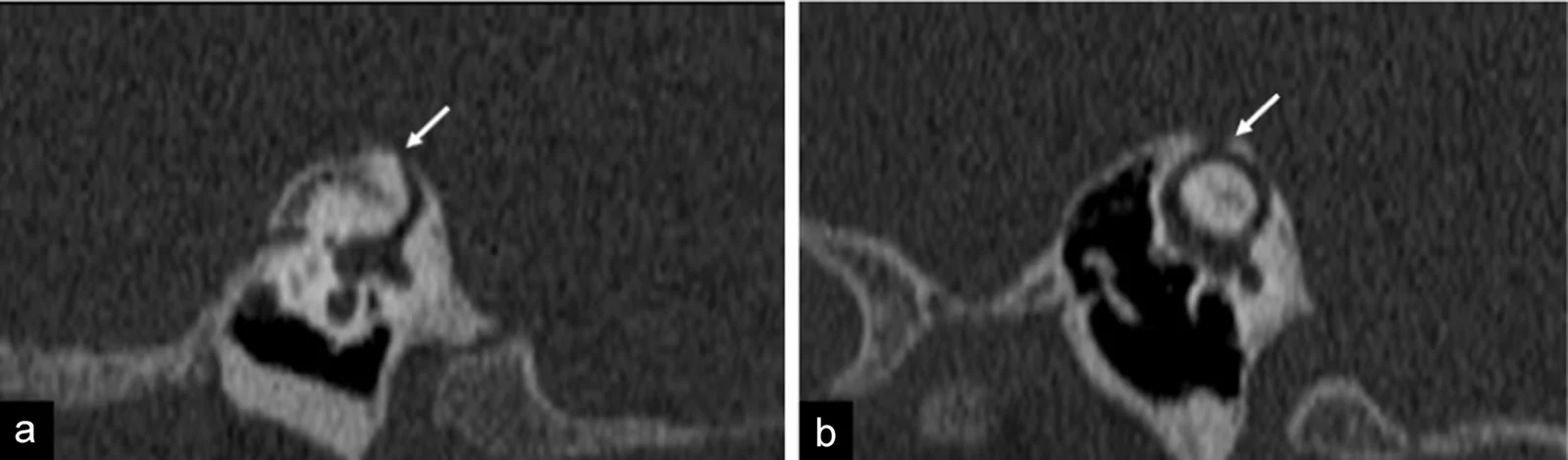
Improved visualization of superior semicircular canal dehiscence using lesion-oriented multiplanar reformations. **a** Coronal temporal HRCT image in a 15-year-old boy presenting with sound-induced vertigo demonstrates limited visualization of the superior semicircular canal (SSC), making confident assessment of dehiscence difficult (*white arrow*). **b** Corresponding Pöschl plane reformation allows visualization of the superior semicircular canal along its entire course and clearly demonstrates focal dehiscence of the canal roof (*white arrow*). This comparison highlights the diagnostic value of lesion-oriented multiplanar reformations for accurate detection of superior semicircular canal dehiscence. *Abbreviations*: *HRCT*, high-resolution computed tomography

Although standard axial and coronal planes form the basis of temporal bone CT evaluation, reliance on these planes alone is insufficient for confident assessment of third-window anomalies. Many inner ear structures—including the semicircular canals, vestibular aqueduct, and cochlear turns—are obliquely oriented, rendering them susceptible to orientation-related artifacts and false-positive or false-negative interpretation on conventional planes [2, 18, 19]. A representative example of orientation-related false-positive appearance on axial CT and its resolution using lesion-oriented MPRs is shown in Fig. 4.

For this reason, CT assessment of suspected third-window anomalies should follow a lesion-oriented reconstruction strategy, in which reformatted images are generated parallel and perpendicular to the specific structure of interest. This approach minimizes partial-volume effects and allows confirmation of suspected bony defects on at least one orthogonal view before establishing a diagnosis of dehiscence [2, 21]. Importantly, this principle applies broadly to evaluation of all potential third-window sites and is not limited to any single anatomic location.

**Fig. 5:**
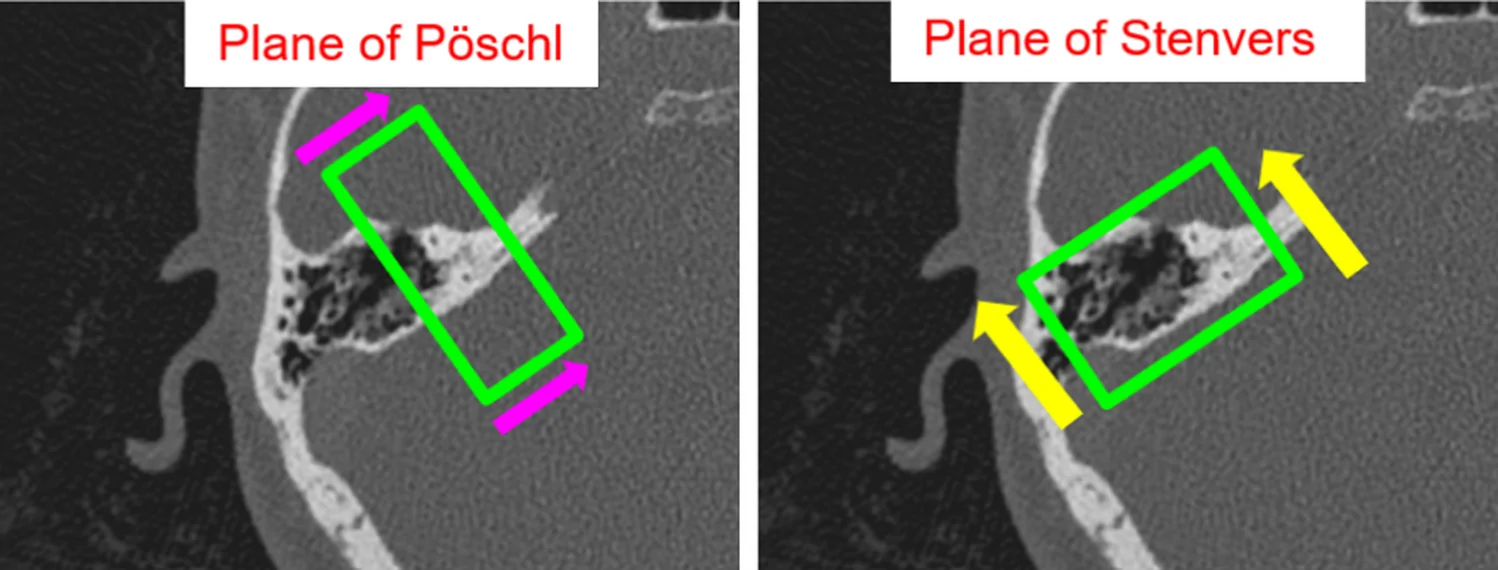
Orientation of Pöschl and Stenvers reconstruction planes on temporal bone CT. Axial temporal bone CT images demonstrating the geometric orientation of the Pöschl and Stenvers reconstruction planes. The Pöschl plane (*left*) is oriented parallel to the long axis of the superior semicircular canal and perpendicular to the petrous apex, providing an optimal short-axis view for evaluation of bony dehiscence. The Stenvers plane (*right*) is oriented perpendicular to the Pöschl plane and parallel to the petrous ridge, offering a complementary long-axis perspective of the bony labyrinth. Colored overlays indicate the relative orientation of each reconstruction plane

In contemporary temporal bone CT practice, oblique reconstructions corresponding to the Pöschl and Stenvers orientations are widely used for evaluation of the superior semicircular canal. The geometric relationship and complementary nature of these reconstruction planes are illustrated in Fig. 5, which demonstrates their respective orientations relative to the long axis of the superior semicircular canal and the petrous ridge. Together, these views provide orthogonal short- and long-axis perspectives that facilitate accurate assessment of the bony canal margins and reduce orientation-related misinterpretation [20, 21].

**Fig. 6:**
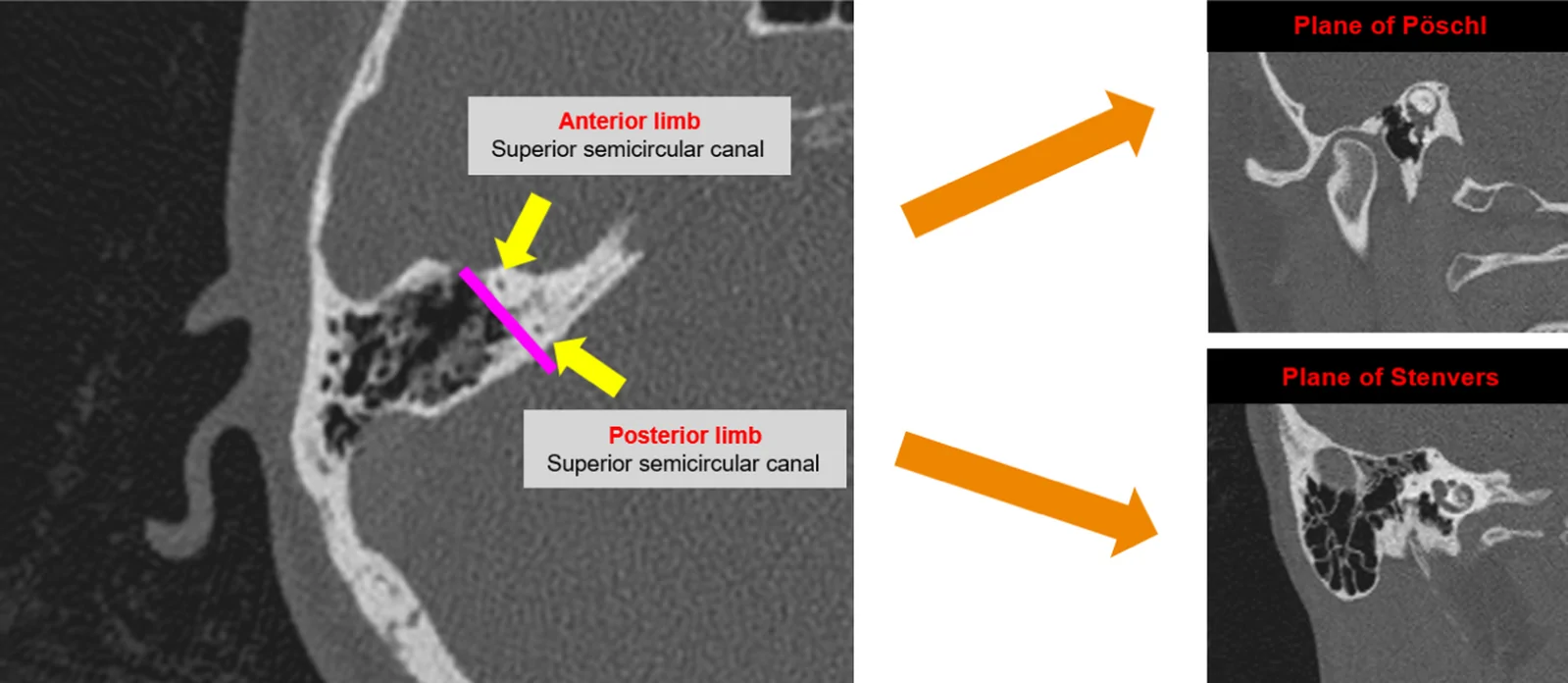
Landmark-based generation of Pöschl and Stenvers views for superior semicircular canal evaluation

Beyond conceptual understanding of plane orientation, accurate generation of lesion-oriented reconstructions requires a reproducible landmark-based approach. Figure 6 demonstrates a practical method for generating Pöschl and Stenvers views using simultaneous visualization of the anterior and posterior limbs of the superior semicircular canal as the key anatomic reference. This landmark-based technique ensures consistent alignment of reformatted images with the true axis of the canal and optimizes detection of subtle bony dehiscence [20, 21].

Axial temporal bone CT image demonstrating the key anatomic landmark for orientation, defined by the simultaneous visualization of the anterior and posterior limbs of the superior semicircular canal. This reference plane serves as the starting point for lesion-oriented multiplanar reconstruction. Pöschl view generated parallel to the superior semicircular canal provides a short-axis perspective optimized for detection of bony dehiscence. Stenvers view is generated perpendicular to the Pöschl plane and aligned with the petrous ridge, providing a complementary long-axis view of the superior semicircular canal.

Several technical and anatomic pitfalls may lead to false-positive or false-negative interpretation of third-window anomalies on CT, particularly in pediatric patients. These include partial-volume averaging, incomplete ossification of the otic capsule, extremely thin but intact bony coverage, and adjacent anatomic variants such as a high-riding jugular bulb. Awareness of these pitfalls and systematic use of lesion-oriented MPRs and optimized bone window settings are essential to ensure diagnostic accuracy and avoid misinterpretation [2, 20]. Common imaging pitfalls and their practical solutions are summarized in Table 4.

**Table 4:** Common imaging pitfalls in CT evaluation of third-window anomalies and recommended solutions

Imaging pitfall	Recommended solution
Partial-volume averaging	Lesion-oriented multiplanar reconstructions
Incomplete otic capsule ossification	Optimized bone window settings ± lesion-oriented multiplanar reconstructions
Extremely thin but intact bone	Bone window optimization and confirmation on orthogonal multiplanar reconstructions ± gray-scale inversion
Adjacent anatomic variants (e.g., high-riding jugular bulb or jugular bulb diverticulum)	Lesion-oriented multiplanar reconstructions

Gray-scale inversion (invert function) may serve as an adjunctive visualization tool during temporal bone CT interpretation [22]. This post-processing display technique reverses image gray-scale values and may improve visualization of subtle osseous abnormalities by enhancing perceived contrast between adjacent structures [22]. Previous observational data have suggested potential utility in temporal bone imaging, particularly for assessment of subtle third-window lesions such as superior semicircular canal dehiscence and cochlear–facial nerve dehiscence, as well as differentiation of extremely thin but intact bone from true dehiscence [22]. Although gray-scale inversion should not replace lesion-oriented multiplanar reconstructions or optimized CT acquisition techniques, it may increase diagnostic confidence in selected equivocal cases [22].

Magnetic resonance imaging (MRI) does not directly depict bony dehiscence and is therefore not sufficient for primary diagnosis of third-window anomalies. However, MRI provides valuable complementary information by demonstrating the fluid-filled inner ear structures and associated labyrinthine abnormalities and is particularly useful for evaluation of enlarged vestibular aqueduct, associated inner ear abnormalities, and coexisting cochleovestibular malformations in pediatric patients with sensorineural hearing loss (SNHL) [2, 13, 14].

Photon-counting detector CT (PCD-CT) has recently emerged as a promising technique for ultra-high-resolution temporal bone imaging and may further improve evaluation of subtle third-window anomalies. Compared with conventional energy-integrating detector CT systems, PCD-CT enables improved spatial resolution and enhanced delineation of fine osseous structures while reducing blooming and partial-volume artifacts [23, 24]. These technical advantages may be particularly valuable for assessing extremely thin otic capsule bone and small dehiscence defects that are susceptible to partial-volume or orientation-related misinterpretation on conventional CT. In addition, the improved dose efficiency of PCD-CT may support radiation dose optimization in pediatric temporal bone imaging while preserving diagnostic image quality [23–25]. Although conventional HRCT remains the current standard in most institutions, PCD-CT is expected to play an increasing role in future temporal bone imaging.

In pediatric patients, CT evaluation of third-window anomalies should strictly adhere to the as low as reasonably achievable (ALARA) principle. Dose optimization strategies include age- and size-adjusted tube voltage and current settings, limitation of scan coverage to the temporal bones, and avoidance of unnecessary multiphase or contrast-enhanced acquisitions [2, 20, 26]. Importantly, dose reduction should not compromise spatial resolution, as inadequate image quality may increase diagnostic uncertainty and lead to repeat examinations, thereby increasing cumulative radiation exposure [2, 20, 26].

## Case-based imaging spectrum of third-window anomalies in children

Third-window anomalies in children demonstrate a broad spectrum of imaging appearances depending on the involved inner ear structure. A case-based imaging approach provides a practical and clinically relevant framework for illustrating these entities and highlighting key radiologic features encountered in pediatric practice.

## Semicircular canal–related third-window anomalies

They are characterized by focal absence of the bony covering of one or more semicircular canals, resulting in abnormal communication between the canal lumen and adjacent structures. Based on the involved canal, these anomalies are classified as superior, posterior, or lateral semicircular canal dehiscence [2, 10–12].

## Superior semicircular canal dehiscence

Superior semicircular canal dehiscence (SSCD) is defined by focal absence of the bony roof overlying the superior semicircular canal, most commonly near the arcuate eminence. In pediatric patients, SSCD is generally considered congenital and may be detected incidentally or during evaluation for auditory or vestibular symptoms [2, 10–12].

**Fig. 7:**
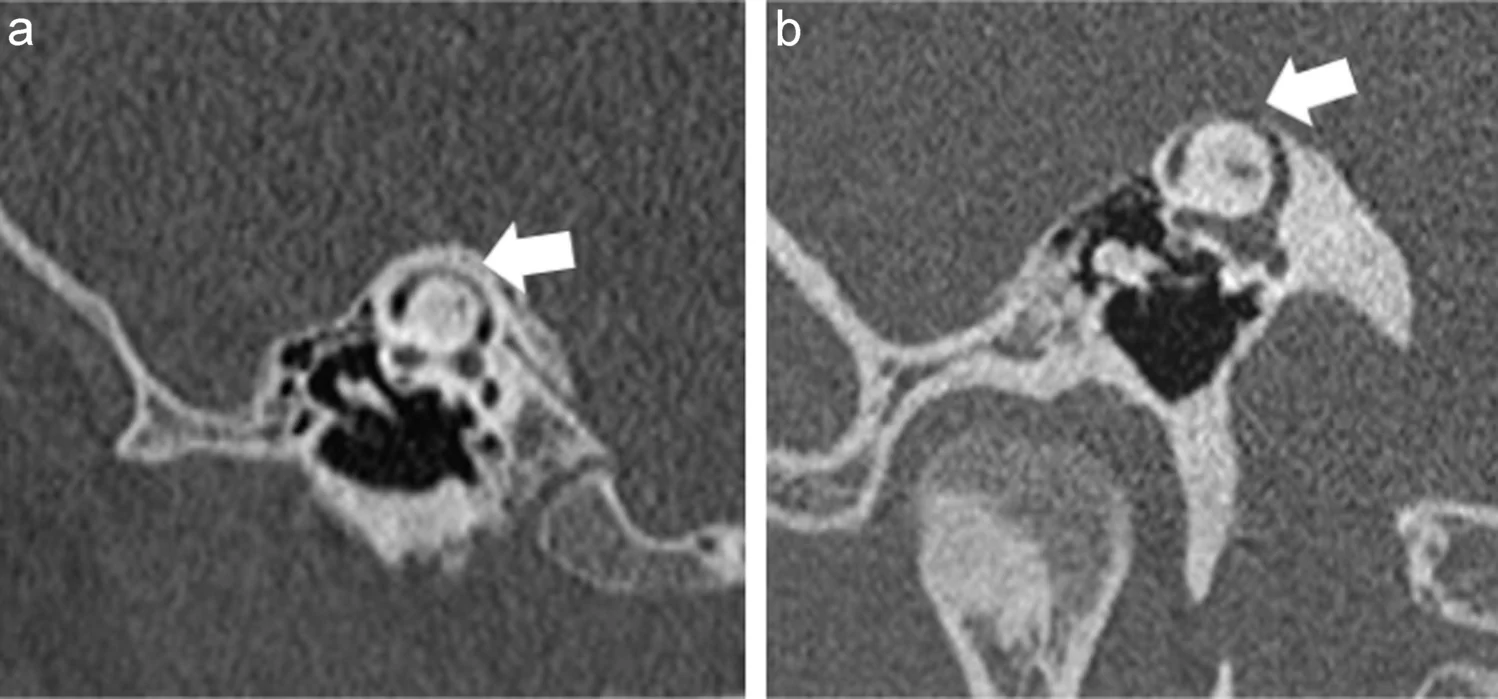
Pöschl plane appearance of the superior semicircular canal: normal versus dehiscent. **a** Pöschl plane reformatted CT image of a 2-year-old girl demonstrating normal bony integrity of the superior semicircular canal. The canal roof is continuously covered by bone without evidence of dehiscence. **b** Pöschl plane reformatted CT image of a 17-year-old boy presenting with dizziness, left aural fullness, and hearing loss, revealing SSCD, characterized by focal absence of the bony covering over the canal. This side-by-side comparison highlights the diagnostic value of Pöschl-oriented reconstructions for differentiating normal anatomy from true dehiscence across pediatric age groups. *Abbreviations*: *SSCD*, superior semicircular canal dehiscence

On CT, a normal pediatric superior semicircular canal demonstrates continuous bony coverage along its roof, whereas true SSCD is characterized by complete focal absence of this bony margin. Figure 7 presents representative CT images from two pediatric patients, illustrating the normal Pöschl-plane appearance of an intact superior semicircular canal and the characteristic imaging findings of SSCD. This side-by-side comparison emphasizes the role of lesion-oriented reconstructions in confidently distinguishing normal anatomy from true dehiscence [12, 27].

## Posterior semicircular canal dehiscence

Posterior semicircular canal dehiscence (PSCD) is less common than superior semicircular canal dehiscence and is frequently associated with adjacent anatomic variants, particularly a high-riding jugular bulb or jugular bulb diverticulum [2, 12, 28]. In pediatric patients, PSCD may present as an isolated canal defect or as part of a broader spectrum of jugular bulb–related abnormalities.

**Fig. 8:**
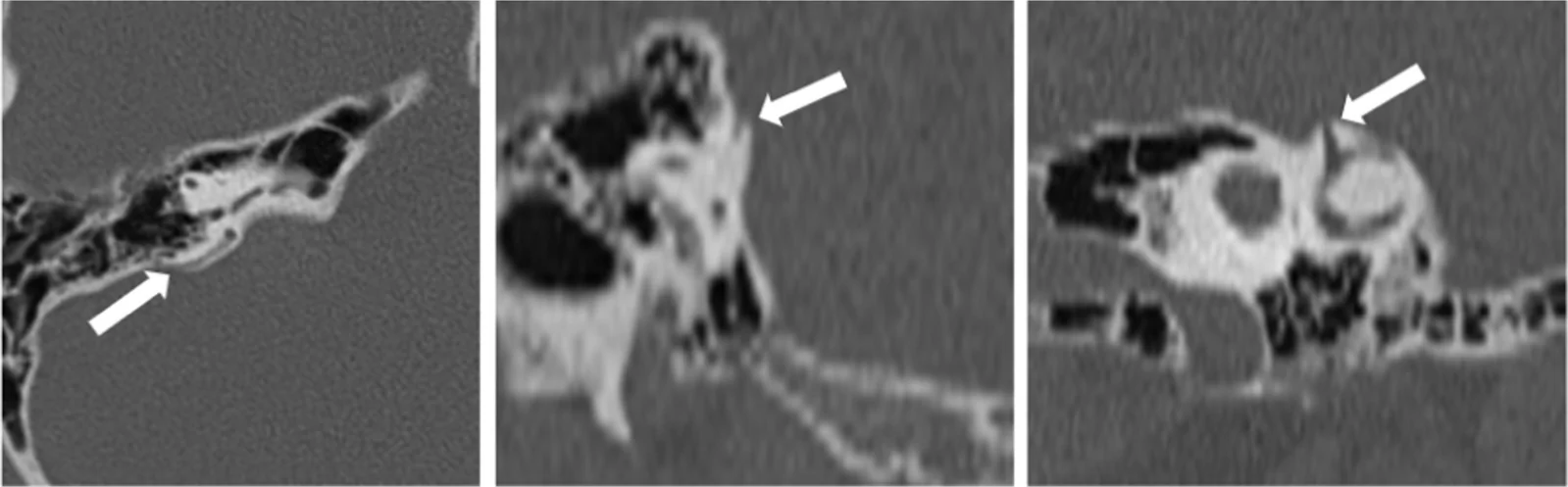
Isolated PSCD in a pediatric patient. Temporal bone CT images with multiplanar reconstructions of an 8-year-old girl presenting with dizziness demonstrate focal absence of the bony wall of the PSC, consistent with PSCD (*white arrows*). Axial (*left*), coronal (*middle*), and sagittal (*right*) views clearly depict the defect, confirming the diagnosis on orthogonal planes. *Abbreviations*: *PSC* posterior semicircular canal, *PSCD* posterior semicircular canal dehiscence

**Fig. 9:**
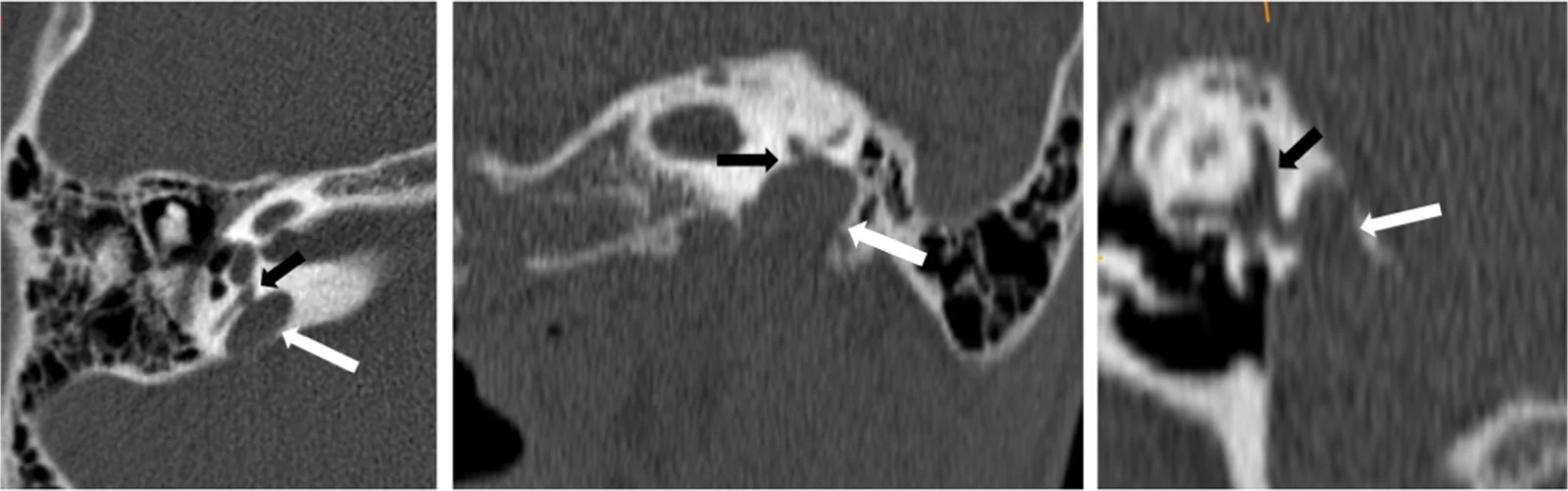
PSC–jugular bulb fistula in a pediatric patient. Temporal bone CT images of a 13-year-old boy presenting with tinnitus and vertigo demonstrate PSCD (*black arrows*) with a fistulous connection to the adjacent jugular bulb (*white arrows*). Axial (*left*), coronal (*middle*), and sagittal (*right*) reconstructions confirm the bony defect and its direct communication with the jugular bulb. *Abbreviations*: *PSC* posterior semicircular canal, *PSCD* posterior semicircular canal dehiscence

On CT, PSCD is characterized by the focal absence of the bony wall of the posterior semicircular canal (PSC). In some cases, progressive erosion or developmental absence of the intervening bone results in direct communication between the PSC and the jugular bulb, forming a posterior canal–jugular bulb fistula. Figures 8 and 9 demonstrate representative pediatric examples of PSCD, including an isolated PSCD and a posterior canal–jugular bulb fistula, highlighting the variable imaging spectrum of this entity [28].

## Lateral semicircular canal dehiscence

Lateral semicircular canal dehiscence (LSCD) is an uncommon semicircular canal–related third-window anomaly and is more frequently encountered as an acquired rather than congenital entity. LSCD is typically associated with chronic otitis media, cholesteatoma, or prior otologic surgery, resulting in erosion of the bony wall of the lateral semicircular canal [2, 12, 28].

On HRCT, LSCD is characterized by focal discontinuity of the bony lateral semicircular canal, often in close proximity to the medially expanding cholesteatoma. Recognition of LSCD is important because this entity may contribute to the third-window–related auditory and vestibular symptoms and has implications for surgical planning, particularly in patients with coexisting middle ear disease or prior otologic interventions [12, 28, 29].

## Vestibular third-window anomalies

Vestibular third-window anomalies arise from abnormal communications between the vestibule and adjacent non-vestibular structures [2, 10]. In pediatric patients, these anomalies are predominantly congenital and commonly present with sensorineural or mixed hearing loss [2, 13].

## Enlarged vestibular aqueduct

**Fig. 10:**
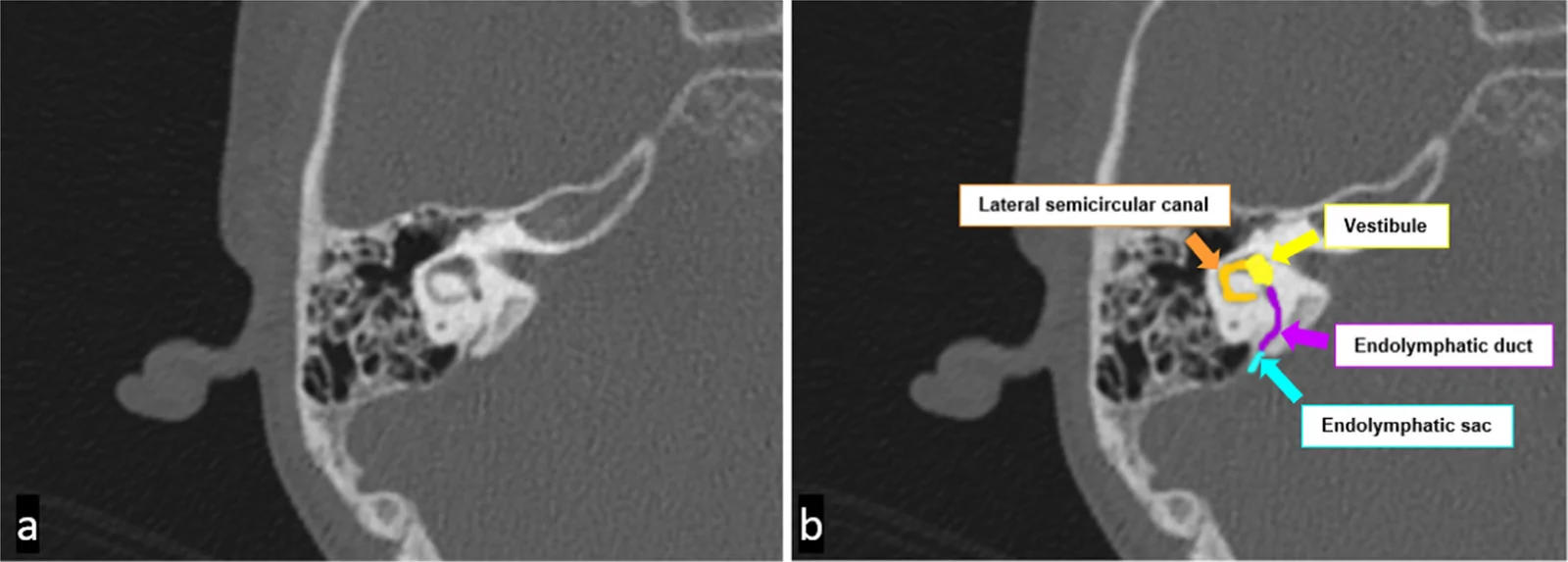
Normal vestibular aqueduct on axial temporal bone CT. Axial high-resolution CT images of the temporal bone demonstrating a normal vestibular aqueduct. **a** Axial CT image showing the normal appearance of the vestibular aqueduct as a thin bony canal extending posteriorly from the vestibule toward the posterior surface of the petrous temporal bone. **b** Corresponding annotated image highlighting the vestibular aqueduct (*colored overlay*), illustrating its anatomic course from the vestibule to the posterior petrous surface. The vestibular aqueduct contains the endolymphatic duct and terminates at the endolymphatic sac

The vestibular aqueduct normally extends from the posterior aspect of the vestibule toward the endolymphatic sac and is visualized on temporal bone CT as a thin bony canal coursing posteriorly from the vestibule [2, 13]. The normal anatomic course of the vestibular aqueduct on axial CT is illustrated in Fig. 10. Enlargement of this structure represents the most common inner ear anomaly in children and a well-established imaging finding in pediatric hearing losses [2, 13].

**Fig. 11:**
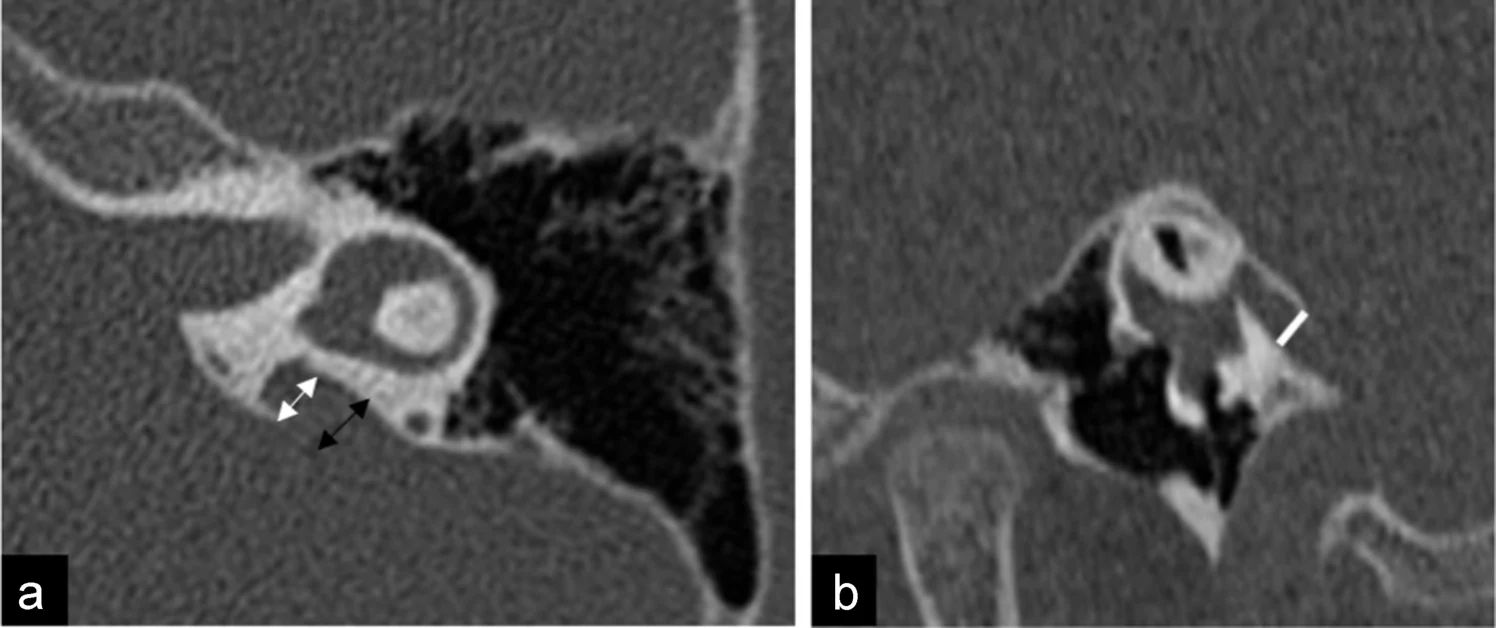
Measurement techniques for enlarged vestibular aqueduct on temporal bone CT of a 2-year-old girl with EVA. **a** Axial CT image demonstrating conventional Cincinnati criteria measurements. The width of the vestibular aqueduct at the operculum measures 3.3 mm (*black arrow*), while the midpoint width measures 2.4 mm (*white arrow*). **b** 45° oblique (Pöschl) reformation aligned with the long axis of the vestibular aqueduct. The midpoint of the vestibular aqueduct is identified, and a perpendicular measurement is obtained along its wall (*white line*), allowing improved visualization of the aqueduct course and more reproducible assessment. *Abbreviations*: *EVA* enlarged vestibular aqueduct

**Fig. 12:**
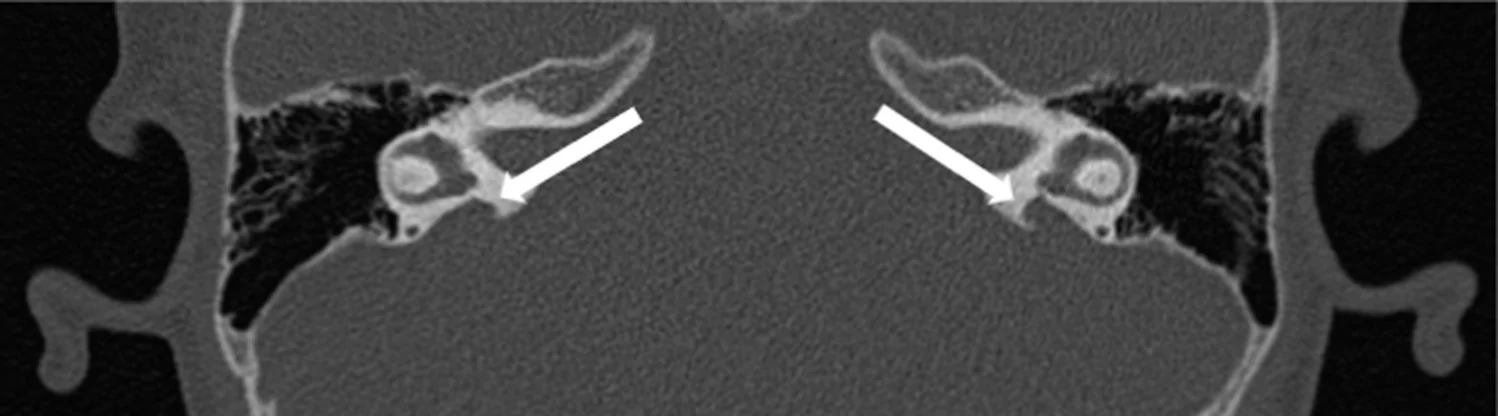
Bilateral EVA in a pediatric patient. Temporal bone CT images of a 2-year-old girl presenting with bilateral SNHL demonstrate bilateral enlargement of the vestibular aqueducts (*white arrows*), consistent with EVA. Symmetric widening of the aqueducts is evident along their posterior course from the vestibule, representing the typical imaging appearance of bilateral EVA in children. *Abbreviations*: *EVA* enlarged vestibular aqueduct, *SNHL* sensorineural hearing loss

Enlarged vestibular aqueduct (EVA) creates an abnormal low-impedance pathway between the vestibule and the endolymphatic sac, thereby modifying normal cochlear and vestibular fluid mechanics [10]. On CT, EVA is characterized by abnormal widening of the vestibular aqueduct along its course. Based on the Cincinnati criteria, a vestibular aqueduct diameter exceeding 0.9 mm at the midpoint or 1.9 mm at the operculum is considered enlarged in the axial plane [29]. More recent studies have emphasized the importance of multiplanar assessment of the vestibular aqueduct, particularly using 45° oblique (Pöschl) reformations aligned with the long axis of the aqueduct [30, 31]. Compared with conventional axial images, evaluation in the Pöschl plane provides improved visualization of the vestibular aqueduct and may improve reproducibility and anatomic accuracy of measurements by better depicting its oblique course [30]. Contemporary CT studies have further demonstrated that the Pöschl plane allows reliable identification of the vestibular aqueduct midpoint and more standardized assessment of enlarged vestibular aqueduct in pediatric patients [31]. Accordingly, lesion-oriented multiplanar reconstructions should be encouraged during CT evaluation of suspected EVA. Representative examples of conventional axial Cincinnati criteria measurements and 45° oblique (Pöschl) reformatted assessment are demonstrated in Fig. 11. Figure 12 demonstrates bilateral EVA in a 2-year-old girl, illustrating symmetric enlargement of the vestibular aqueducts and the typical imaging appearance of bilateral EVA in pediatric patients. EVA may coexist with additional cochleovestibular malformations, including cochlear anomalies such as Mondini dysplasia, which may contribute to hearing impairment [32].

**Fig. 13:**
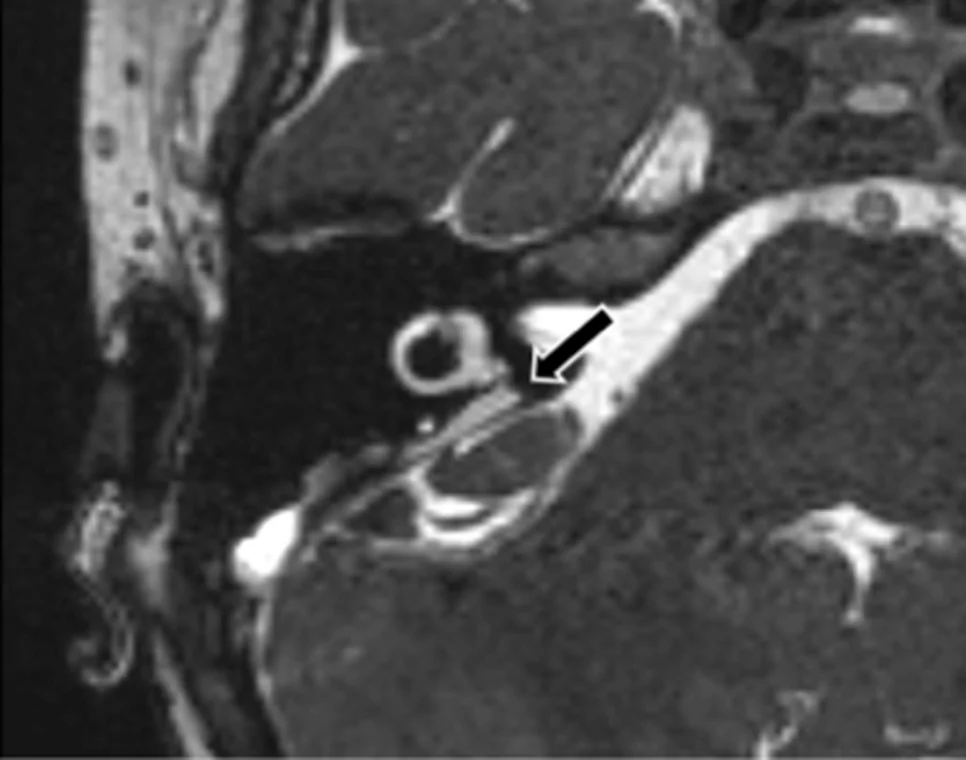
EVA on temporal MRI of a pediatric patient. Temporal MRI of a 2-year-old girl presenting with SNHL demonstrating enlarged right vestibular aqueduct (*black arrow*), visualized as increased fluid signal intensity of the endolymphatic duct and sac, consistent with vestibular third-window pathology. *Abbreviations*: *EVA* enlarged vestibular aqueduct, *MRI* magnetic resonance imaging, *SNHL* sensorineural hearing loss

In addition to CT, MRI may provide complementary information in the evaluation of EVA by directly demonstrating the endolymphatic sac and duct. On MRI, EVA appears as enlargement of the endolymphatic duct and sac with increased fluid signal intensity, which may be more conspicuous than the corresponding bony changes on CT, particularly in young children [2, 13, 14]. Figure 13 illustrates this complementary role of MRI in a 2-year-old girl, in whom temporal MRI clearly demonstrates enlargement of the right vestibular aqueduct.

## Inner ear malformations causing a dehiscence between the vestibule and non-vestibular sites

In some congenital inner ear malformations, enlargement of the vestibular aqueduct may coexist with additional bony defects that create a direct communication between the vestibule and adjacent non-vestibular structures, functioning as multiple or complex third windows [2, 10, 14]. These combined anomalies reflect more extensive developmental disturbances of the otic capsule and may result in more pronounced alterations of inner ear fluid mechanics [10, 14].

**Fig. 14:**
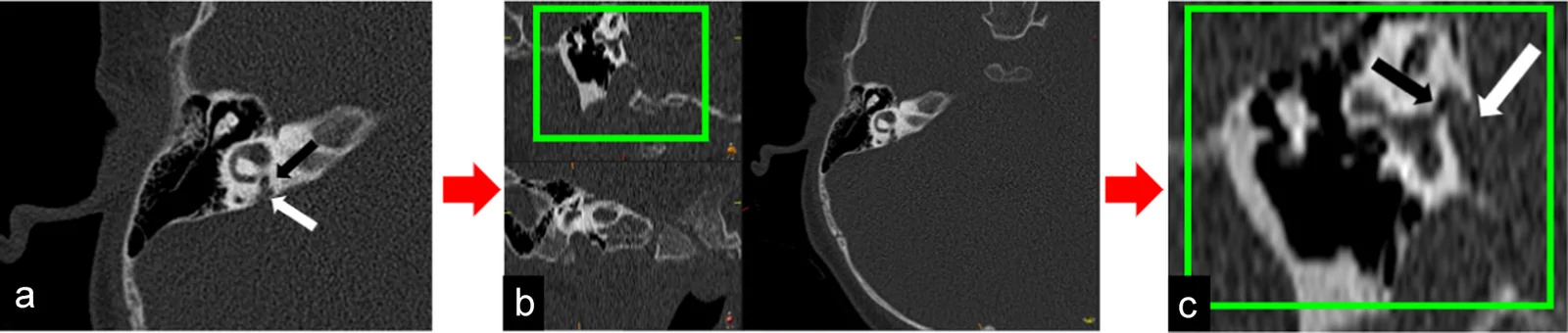
Enlarged vestibular aqueduct with abnormal vestibule–non-vestibular communication. **a** Axial temporal bone CT image of a 21-month-old girl with suspected hearing loss shows an enlarged right vestibular aqueduct (*white arrow*) in close anatomic relationship with the PSC (*black arrow*). **b** MPR of the same case demonstrates the course of the enlarged vestibular aqueduct, allowing improved appreciation of its spatial orientation and relationship to the PSC. **c** Coronal MPR image reveals that the enlarged vestibular aqueduct (*white arrow*) opens toward the PSC (*black arrow*), establishing an abnormal vestibule–non-vestibular communication and highlighting the importance of lesion-oriented MPRs for accurate anatomic delineation. *Abbreviations*: *MPR* multiplanar reconstruction, *PSC* posterior semicircular canal

Figure 14 illustrates a representative pediatric case in which an enlarged vestibular aqueduct demonstrates a close anatomic relationship with the PSC. Axial images show the enlarged vestibular aqueduct adjacent to the PSC, while coronal multiplanar reconstructions confirm that the aqueduct opens toward the canal, establishing an abnormal vestibule–non-vestibular communication. This example underscores the importance of lesion-oriented MPRs in accurately delineating complex anatomic relationships in vestibular third-window anomalies.

## Cochlear third-window anomalies

Cochlear third-window anomalies arise from defects of the otic capsule involving the cochlea, resulting in abnormal low-impedance communications between the perilymphatic space and adjacent structures. These defects alter normal cochlear pressure transmission and may lead to hearing loss, vestibular symptoms, or clinical presentations that mimic other inner ear disorders [1, 2, 10].

## Dehiscence between the cochlea and non-cochlear sites

In pediatric patients, cochlear dehiscence may occur secondary to inner ear malformations or traumatic injury, resulting in abnormal communication between the cochlea and adjacent non-cochlear structures, such as the facial nerve canal or carotid canal. These entities are referred to as cochlear–facial or cochlear–carotid dehiscence, respectively [2, 14, 15]. These defects function as third windows by allowing abnormal fluid shunting away from the cochlea.

**Fig. 15:**
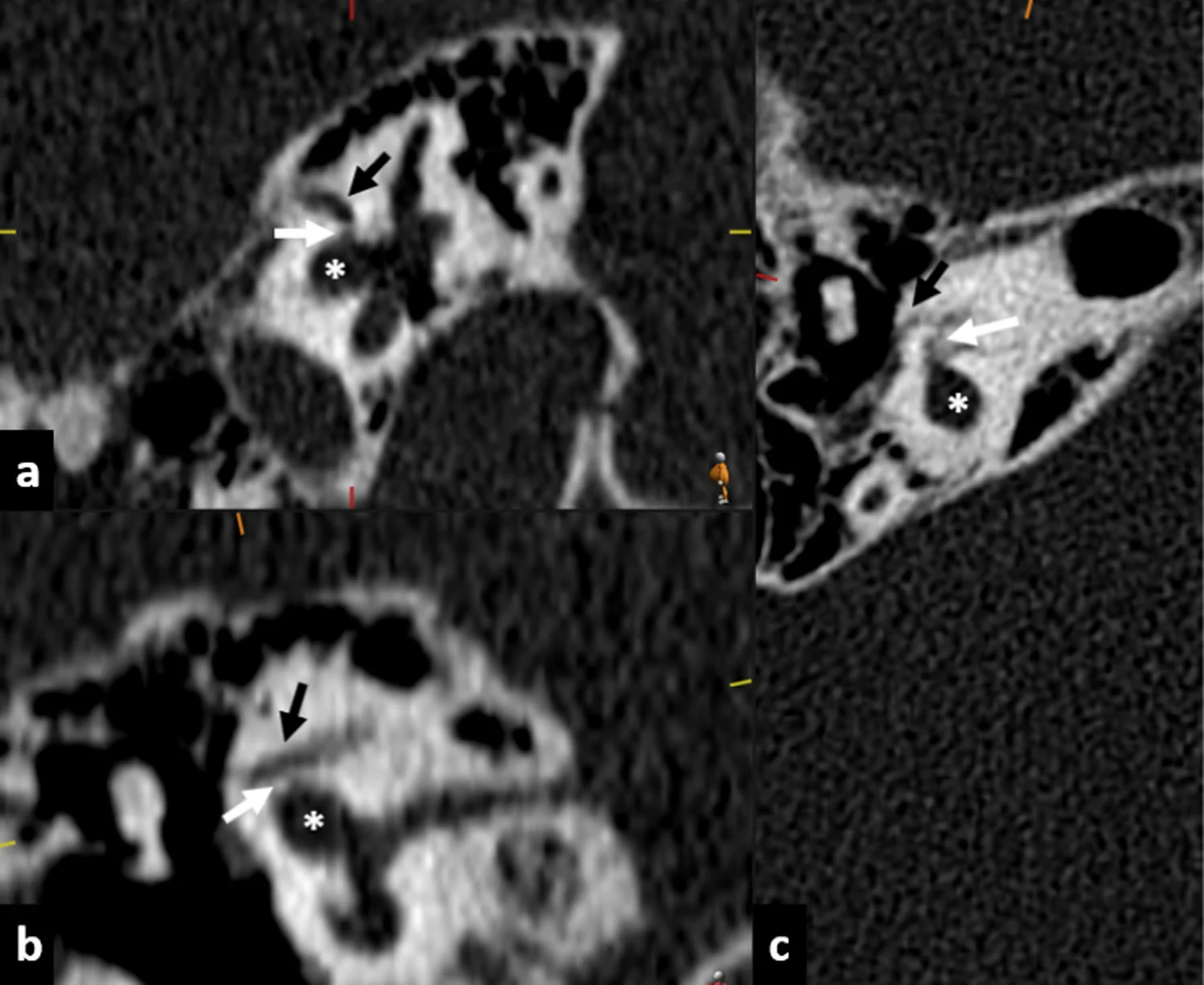
Congenital cochlear–facial dehiscence in a pediatric patient. Multiplanar temporal bone CT images of a 7-year-old boy presenting with near-total right-sided hearing loss. Coronal (**a**), sagittal (**b**), and axial (**c**) MPR images demonstrate the cochlear basal turn (*white asterisk*), a developmental osseous defect creating a third-window connection (*white arrow*), and the tympanic segment of the facial nerve canal (*black arrow*), consistent with congenital cochlear–facial dehiscence. *Abbreviations*: *MPR*, multiplanar reconstruction

Figure 15 demonstrates a representative congenital pediatric case showing cochlear–facial dehiscence, characterized by a developmental osseous defect extending from the cochlear basal turn toward the tympanic segment of the facial nerve canal, consistent with a congenital cochlear third-window anomaly.

**Fig. 16:**
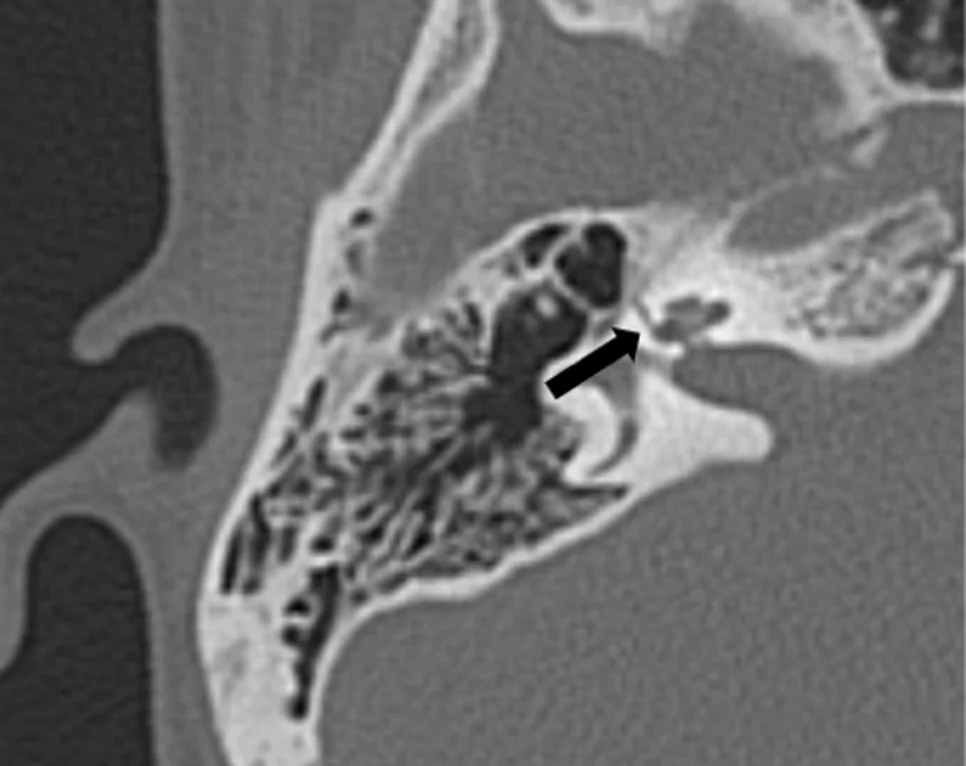
Traumatic cochlear dehiscence with extension to the facial nerve canal. Axial thin-section temporal bone CT image of a 12-year-old girl with right-sided profound hearing loss and a remote history of fall-related head trauma demonstrates a nondisplaced transverse fracture (*black arrow*) involving the cochlea with extension into the facial nerve canal, consistent with cochlear dehiscence. This abnormal communication represents a traumatic cochlear third window

Figure 16 demonstrates a representative pediatric case in which a nondisplaced transverse temporal bone fracture extends through the cochlea toward the facial nerve canal, creating an abnormal post-traumatic communication. Although otic capsule fractures frequently produce hearing loss through direct injury to internal labyrinthine structures, persistent osseous defects may also contribute to acquired cochlear third-window physiology in selected cases [15].

## Dehiscence between the internal auditory canal and the scala vestibuli

A distinct group of cochlear third-window anomalies involves abnormal communication between the internal auditory canal (IAC) and the scala vestibuli. This entity is classified as IP-3 anomaly and is classically associated with X-linked deafness (DFNX2) and reflects a congenital defect of the otic capsule related to abnormal embryologic development [33, 34].

**Fig. 17:**
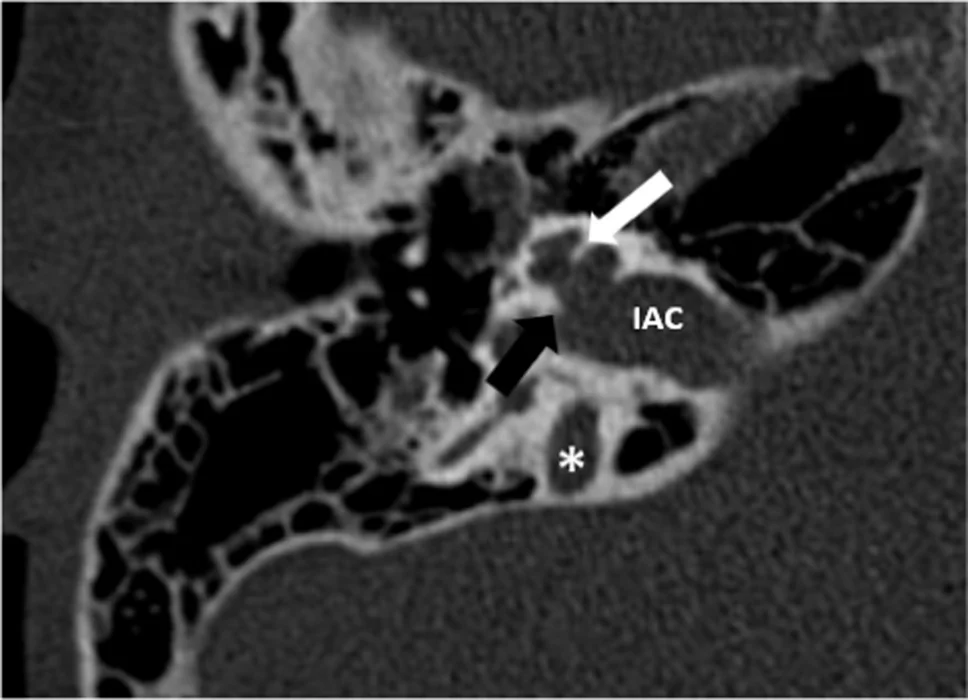
Cochlear–internal auditory canal dehiscence in X-linked deafness (IP-3 anomaly). Temporal bone CT images of a 6-year-old boy demonstrate classic imaging features. The cochlea shows absence of the modiolus with preservation of the interscalar septa (*white arrow*), creating a characteristic “corkscrew” appearance. The cochlear aperture is widened (*black arrow*), and the internal auditory canal (IAC) is enlarged. An abnormally enlarged right vestibular aqueduct is also present (*white asterisk*), representing an associated third-window anomaly

On CT, characteristic imaging findings include absence of the modiolus, preservation of the interscalar septa resulting in a characteristic “corkscrew” appearance of the cochlea, widening of the cochlear aperture, and enlargement of the internal auditory canal [2, 33]. Figure 17 illustrates these classic features in a pediatric patient. Associated inner ear abnormalities, such as enlarged vestibular aqueduct, may coexist and further contribute to third-window physiology [13, 33]. Recognition of cochlear–IAC dehiscence is clinically important, as this anomaly represents a true third window and has implications for surgical planning, particularly in pediatric patients considered for cochlear implantation [2, 33].

## Conclusion

Third-window anomalies in children encompass a diverse group of inner ear disorders unified by a common pathophysiologic mechanism but distinguished by their anatomic location and imaging appearance. High-resolution temporal bone CT, interpreted using lesion-oriented multiplanar reconstructions, provides a robust framework for accurate diagnosis. Familiarity with the pediatric spectrum of third-window anomalies and their characteristic imaging features enables confident radiologic assessment and supports appropriate clinical management.

## Data Availability

The datasets generated and/or analyzed during the current study are not publicly available due to patient privacy and ethical restrictions but are available from the corresponding author upon reasonable request.
